# Identifying mental health status using deep neural network trained by visual metrics

**DOI:** 10.1038/s41398-020-01117-5

**Published:** 2020-12-14

**Authors:** Somayeh B. Shafiei, Zaeem Lone, Ahmed S. Elsayed, Ahmed A. Hussein, Khurshid A. Guru

**Affiliations:** 1 Applied Technology Laboratory for Advanced Surgery (ATLAS), Roswell Park Comprehensive Cancer Center, Buffalo, NY USA; 2 Department of Urology, Roswell Park Comprehensive Cancer Center, Buffalo, NY USA

**Keywords:** Human behaviour, Diseases

## Abstract

Mental health is an integral part of the quality of life of cancer patients. It has been found that mental health issues, such as depression and anxiety, are more common in cancer patients. They may result in catastrophic consequences, including suicide. Therefore, monitoring mental health metrics (such as hope, anxiety, and mental well-being) is recommended. Currently, there is lack of objective method for mental health evaluation, and most of the available methods are limited to subjective face-to-face discussions between the patient and psychotherapist. In this study we introduced an objective method for mental health evaluation using a combination of convolutional neural network and long short-term memory (CNN-LSTM) algorithms learned and validated by visual metrics time-series. Data were recorded by the TobiiPro eyeglasses from 16 patients with cancer after major oncologic surgery and nine individuals without cancer while viewing18 artworks in an in-house art gallery. Pre-study and post-study questionnaires of Herth Hope Index (HHI; for evaluation of hope), anxiety State-Trait Anxiety Inventory for Adults (STAI; for evaluation of anxiety) and Warwick-Edinburgh Mental Wellbeing Scale (WEMWBS; for evaluation of mental well-being) were completed by participants. Clinical psychotherapy and statistical suggestions for cutoff scores were used to assign an individual’s mental health metrics level during each session into low (class 0), intermediate (class 1), and high (class 2) levels. Our proposed model was used to objectify evaluation and categorize HHI, STAI, and WEMWBS status of individuals. Classification accuracy of the model was 93.81%, 94.76%, and 95.00% for HHI, STAI, and WEMWBS metrics, respectively. The proposed model can be integrated into applications for home-based mental health monitoring to be used by patients after oncologic surgery to identify patients at risk.

## Introduction

Cancer significantly affects the quality of life of patients. Psychologic evaluation and support of patients are key to alleviate emotional distress, enhance coping, and improve the ability of handling cancer diagnosis, subsequent management, and overall prognosis^1,2^. It has been shown that the extent of disease and physical impairment from treatment is associated with the severity of mood disorders among patients with lung cancer^3^. Moreover, the frequency of intrusive thoughts in many patients with cancer diagnosis, especially breast cancer survivors, is primarily related to psychological distress^4^. One study found that health metrics are associated with behavioral and psychological changes, and reducing psychological distress was crucial for better health^2^. Among patients with pancreatic cancer, 71% had symptoms of depression and 48% had anxiety-related disorders^5^. A strong association between suicidal ideation and depression in patients with advanced cancer has been reported, and the incidence of suicide in patients diagnosed with cancer is approximately double the incidence in the general population^6,7^.

Despite the general agreement about the importance of psychological assessment and/or intervention for patients with malignancy, there is lack of an objective method for evaluation of the mental health of this patient cohort^8^. Currently, mental health evaluation is performed by self-reported, subjective, questionnaire-based evaluation that may be time consuming and complicated^9–12^. One form included 65 items, and a shorter version was developed to facilitate administration among patients on cancer therapies that included 37 items^9–11,13^. Another utilized self-reported Patient Health Questionnaire-9 (PHQ-9) compound scores, based on typing patterns to detect tendency towards depression^14^.

The development of an objective mental health evaluation method that automatically monitors mental health metrics can improve and promote mental health evaluation, streamline referrals to psychosocial services, and improve patient care.

Some of the studies proposing objective methods for mental health evaluation summarized in Table 1. However, the majority of these studies propose methods that require feature engineering and also none of them takes into account a clinically approved assessment method.

**Table 1:** Some of existing algorithms employed for objective mental health metrics evaluation.

Author	Algorithm	Accuracy	Data	Task	Subjects	Mental health metric	Number of classes
Yamada et al.^37^	Support vector machine	91%	Visual features	Watching video	18	Mental fatigue	2
Zhai et al.^67^	Naive Bayes algorithm, decision tree classifier, and SVM	90%	Eye gaze, skin temperature, blood volume	Computer game	32	Stress	2
Wu et al.^39^	Naive Bayes algorithm	85%	Eye-tracking measures	Robotic skills simulation tasks	8	Mental workload	2
Alghowinem et al.^68^	Feature fusion + Support Vector Machine	88%	Speaking behavior, eye activity, and head pose	Verbal and non-verbal behavior	60	Depression	2

Eye movements are a function of brain activity and extra-ocular muscle properties^15^. Motor function of the eye is linked to the central nervous system, therefore, disorders that affect the cerebral cortex, the brainstem, or the cerebellum can disturb ocular motor function^16–19^. Visual perception demands proper functioning of ocular motor systems which control the position and movement of eyes (to focus on corresponding areas of the retinas of both eyes^20^), in addition to pupil size adjustment. Human perception of the environment relies on the capacity of brain neural networks (especially within the visual cortex) to adapt to changes in stimuli^21–24^. Understanding the rate of adaptation to stimuli by cortical networks is essential to understand the relationship between sensory coding and behavior^21–24^. Prior studies have confirmed a strong association between ocular motor function and cognitive and mental disorders, including Alzheimer, Parkinson, Huntington and Wilson’s diseases^17,25–30^, in addition to psychiatric disorders such as autism^31,32^, attentional disorder^18^, antisocial personality disorder^33^, and post-traumatic stress disorder^34,35^. This relationship has been shown in experimental psychology and clinical neuroscience. It has already been shown that inhibitory saccades are impaired in Alzheimer’s disease, and is attributed to neurodegeneration in the frontal and prefrontal lobes^17,29,30^. Prior studies have also shown that after an unpleasant-stimulus, emotions were indexed by eye-blink startle, and left-sided frontal EEG activation occurred^36^. Visual metrics have also been proposed for detection of human physiological changes including mental fatigue^37^, cognition and cognitive development^38^, mental workload evaluation^39,40^, stress level in students^41^, threat-related emotional facial expressions in infancy^42^, shared attention for virtual agent^43^, and emotional arousal and autonomic activation^44^.

Deep learning is a set of training methods that allows automatic processing of inputs without feature engineering via hierarchical layers^24,25^. Due to its superiority in complex pattern recognition, deep learning has become state of the art, and has achieved success in areas such as strategic games^26^, speech recognition^27^, medical imaging^28^, and health informatics^29^. However, data about deep learning techniques for the evaluation of mental health remains scarce.

Combination of CNN and LSTM techniques has been frequently applied to physiological parameters for diagnosis, detection, and monitoring of health in several medical applications^45–47^. The LSTM technique is a special form of recurrent neural network (RNN)^48^. It is well-suited for the classification of time-series signals^49^. On the other hand, CNN technique is known for extracting the significant features in data^50^.

In this study, we investigate the feasibility of using deep learning and developing algorithm to utilize visual metrics for objective evaluation of mental health.

## Methods

### Subjects

Sixteen patients who underwent an oncologic surgical procedure at Roswell Park Comprehensive Cancer Center and nine volunteers without cancer were included (I 43217, NCT03688945). Procedures included: gastrointestinal (46%: gastrectomy, HIPEC, colectomy, and Whipple), urologic (35%: radical cystectomy, radical nephrectomy, and parastomal hernia repair), thoracic (13%: minimally invasive esophagostomy), and soft tissue procedures (7%, amputation). Patients did not require continuous monitoring and had no visual impairment. Informed consent was obtained from all participants.

### Experimental setup

All participants (*n* = 25) attended at least one session for 15 min at the dedicated in-house art gallery, developed in collaboration with the Albright Knox Art Gallery. Eighteen pieces of art were selected based on the suggestions within the existing literature, by an expert panel of art professionals from the Albright-Knox Art Gallery^51–53^. All participants looked at each specific art for 50 s and they look at 18 art works in the same order. Only time portions that the subject is directly looking at each artwork are included in the analyses.

Art selection was guided by the Albright-Knox’s mission-philosophy of providing inspirational spaces that support learning and inquiry. Art that rewards multiple or prolonged viewing were selected in order to hold the viewer’s attention. Compositions and content interpreted as uplifting, positive or transcendent were also considered.

**Fig. 1:**
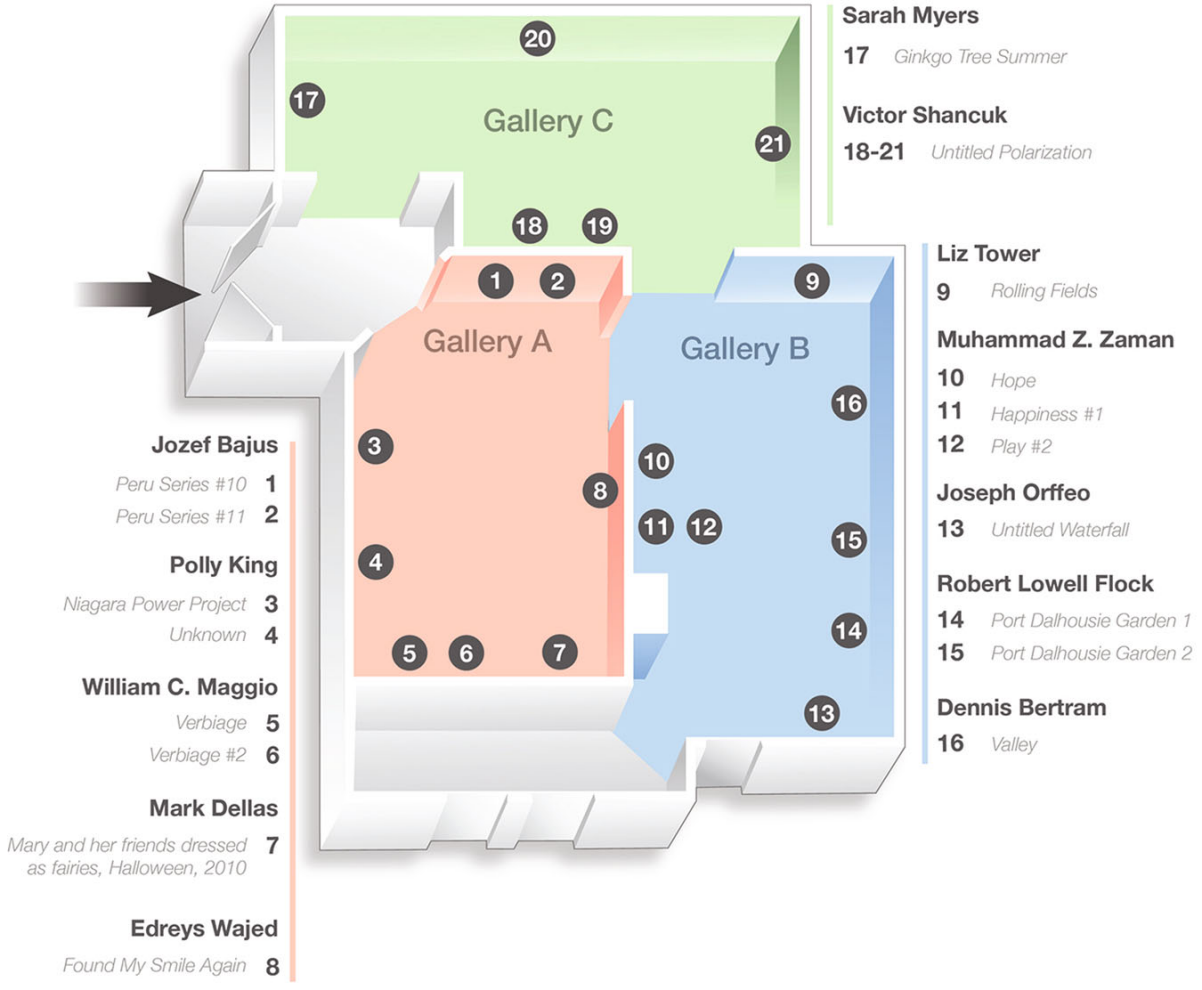
Art gallery layout and artwork. Gallery A includes ‘abstract’ art types. Gallery B includes ‘figuration’ art types, and gallery C includes ‘landscape’ art types.

Selected artwork included three categories: abstract, figurative, and landscape (Fig. 1). Based on Getty’s Art and Architecture Thesaurus, abstract art referred to works that were not clear representations of objectives from reality and instead rely on shapes, colors, forms, and gestures to achieve their effects. Figurative featured representation of a form or figure generally that of human or animal and retained ties to the visible world. Landscape art depicted outdoor scenes typically dominated by the land, hills, fields, and other natural elements. The art gallery was divided into three spaces; each corresponds to the specific type of art included. Patients and participants were blinded to the type of art.

TobiiPro2 eyeglasses were used to record eye movement time-series metrics (sampling rate: 100 Hz) from participants while they viewed the art in the gallery. The recorded time series include 20 features of time, gaze point X, gaze point Y, gaze point 3D X, gaze point 3D Y, gaze point 3D Z, gaze direction left X, gaze direction left Y, gaze direction left Z, gaze direction right X, gaze direction right Y, gaze direction right Z, pupil position left X, pupil position left Y, pupil position left Z, pupil position left X, pupil position left Y, pupil position left Z, pupil diameter left, and pupil diameter right. Total recordings were 370, and the duration of each recording was 15 min.

### Participant-based evaluations

All participants completed three validated post-study questionnaires assessing hope (HHI; Herth Hope Index), anxiety (STAI; State-Trait Anxiety Inventory for Adults), and mental wellbeing (WEMWBS; Warwick-Edinburgh Mental Wellbeing Scale).

*HHI (range 12–48)* is a validated 12-item scale with 4 response categories evaluating the level of hope. Higher HHI scores indicated higher hope level^54^. Scores were categorized into class 0 (low) (12 < HHI < 36), class 1 (intermediate) (36 < HHI < 42), and class 2 (high) (42 < HHI < 48) levels of hope^55,56^.

*STAI (range 20–80)* is a 20-item scale with four response categories. Higher values indicate higher anxiety. Based on psychotherapy literature, we considered 20 < STAI < 44 as class 0 (normal), 44 < STAI < 54 as class 1 (risk of anxiety and suggests a mood disorder), and 54<STAI < 80 as class 2 (significant symptoms of anxiety)^57^.

*WEMWBS (range 14–70):* it is 14-item scale with five response categories covering functioning aspects of mental well-being. Three categories were considered for WEMWBS, class 0: 14 < WEMWBS < 42 (low), class 1: 42 < WEMWBS < 59 (intermediate), and class 2: 59 < WEMWBS < 70 (high)^58,59^.

### Visual data preprocessing

Tobii Pro eye glasses2 are an infrared video-based remote eye-tracking system used to record visual time series at 100 Hz. The moving average filter was applied to gaze data for noise reduction, while the window size of three points was considered.

### Deep neural network

Our mental health metrics (HHI, STAI, WEMWBS) evaluation method was formalized as a supervised three-class classification problem. The Inputs to the model were extracted by using multivariate visual time series. The output of the model was the predicted labels representing corresponding mental health metrics levels of individuals, which can be encoded as class ‘0’, class ‘1’, class ‘2’. The ground-truth mental health metrics were acquired from participants’ subjective assessments and clinical cutoff scores. The objective cost function for training the network was defined as a categorical cross-entropy cost, to train CNN-LSTM to output a probability over the three classes for each input.

#### Network architecture

The architecture of our proposed deep neural network consists of 1D convolution, dropout, maxpooling, flatten, lstm, fully connected, and softmax (Table 2). The depth of the network was chosen after trial-and-error from the training/validation procedure.

**Table 2:** Deep neural network architecture.

Layers
Time_distributed, Conv1D, Filters: 64, kernel size: 3, activation: ‘ReLU’
Time_distributed, Conv1D, Filters: 64, kernel size: 3, activation: ‘ReLU’
Time_distributed, Dropout (0.5)
Time_distributed, MaxPooling1D (pool_size = 2)
Time_distributed (Flatten)
LSTM (100)
Dropout (0.5)
Dense, size: 100, activation = ’ReLU’
Dense, size: 3, activation: ‘softmax’

The CNN-LSTM architecture, an LSTM architecture specifically designed for sequence prediction problems, involves using Convolutional Neural Network (CNN) layers for feature extraction on input data combined with LSTMs to support sequence prediction^60^. The CNN LSTM model will read subsequences of the main sequence (time series) in as blocks/wraps, extract features from each block, then allow the LSTM to interpret the features extracted from each block^45^.

#### Input

We used the aforementioned recorded visual metrics to develop inputs to deep neural network architecture. Visual metrics recorded from 16 patients and 9 volunteers without cancer were used to train the model and tune deep neural network parameters. *Data Augmentation*: Limited size of the available dataset might cause overfitting of the deep model^61^. To prevent happening this problem, we applied data augmentation techniques to time-series data to enlarge the size of the dataset and increase classification accuracy^62–64^. Before inputting data into our network, we carried out a label-preserving cropping with a sliding window, where the motion sub-sequences were extracted using a sliding window with a fixed-size, within the trial. The annotation for each window is identical to the class label of the original trial, from which the sub-sequences are extracted. We considered a time window moving throughout 20 considered visual time series to extract blocks. One approach to implementing this model was to split each window of 51-time points into subsequences for the CNN model to process. The 51-time points in each window were split into three subsequences of 17-time points by wrapper layer. We then considered sequences with a length of 17-time points and 20 features as input to the CNN-LSTM algorithm.

#### Implementation

**Fig. 2:**
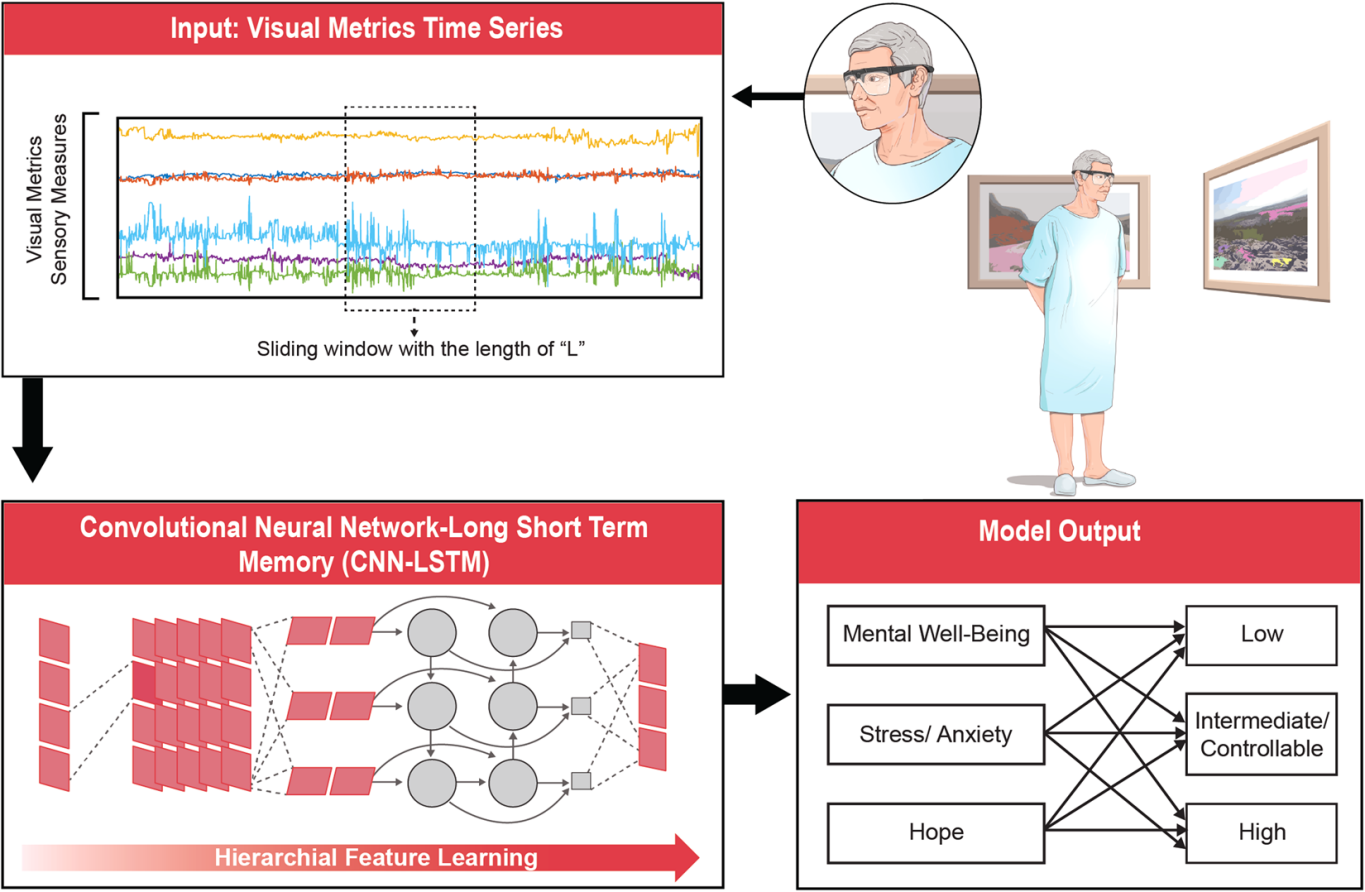
Overview of developed model for objective evaluation of mental health status. Three different networks with the same architecture are trained to evaluate HHI, STAI, and WEMWBS metrics. The HHI, STAI, and WEMWBS are metrics used for subjective evaluation of hope, stress/anxiety, and mental well-being, respectively. Blocked data through visual metrics time series were used as input to the CNN-LSTM model. In this study, sliding window length ‘L’ is 51 and 20 visual metrics are considered as dimension of input to the network. The output of the model is level of mental well-being (evaluated by WEMWBS metric), anxiety/stress (evaluated by STAI metric and hope (evaluated by HHI metric) based on three categories of low (class label: 0), intermediate (class label: 1), and high (class label: 2) categories.

A Convolutional neural network-Long short-term memory (CNN-LSTM) algorithm was implemented using Keras library with Tensorflow backend based on Python 3.6. During the optimization, our network was trained by minimizing the categorical cross-entropy cost between the predicted and ground-truth labels. To train the net efficiently, we run mini-batch with the size of 64 to update gradient descent, which calculated network parameters on a subset of the training data at each iteration^65^. For training, totally 250 epochs were run, and the network parameters were optimized by Adam optimizer^66^, and parameters of learning rate = 0.0001, beta1 = 0.9, beta2 = 0.999, epsilon = 1 × 10^−8^. Overview of the developed model is represented in Fig. 2. Networks with the same architecture are trained to evaluate HHI, STAI, and WEMWBS metrics.

To validate the model classification, we used leave-one-supertrial-out (LOSO) cross-validation schemes in this work. This process is repeated in five fold where each fold consists of each one of the five supertrials. The average of all five-fold performance measures in each test set is reported and gives classification results.

## Results

**Fig. 3:**
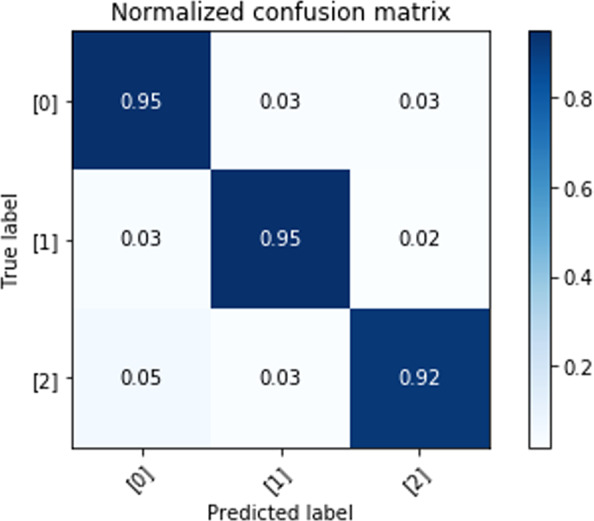
Normalized confusion matrix for HHI evaluation utilizing visual metrics and CNN-LSTM algorithm. Element value (m, n) of this matrix, and color, represent the probability of predicted HHI level n, given the ground-truth HHI level m, where m and n have labels of 0 (low), 1(intermediate/controllable), and 2 (high). The diagonal elements of this matrix correspond to correct predictions.

**Fig. 4:**
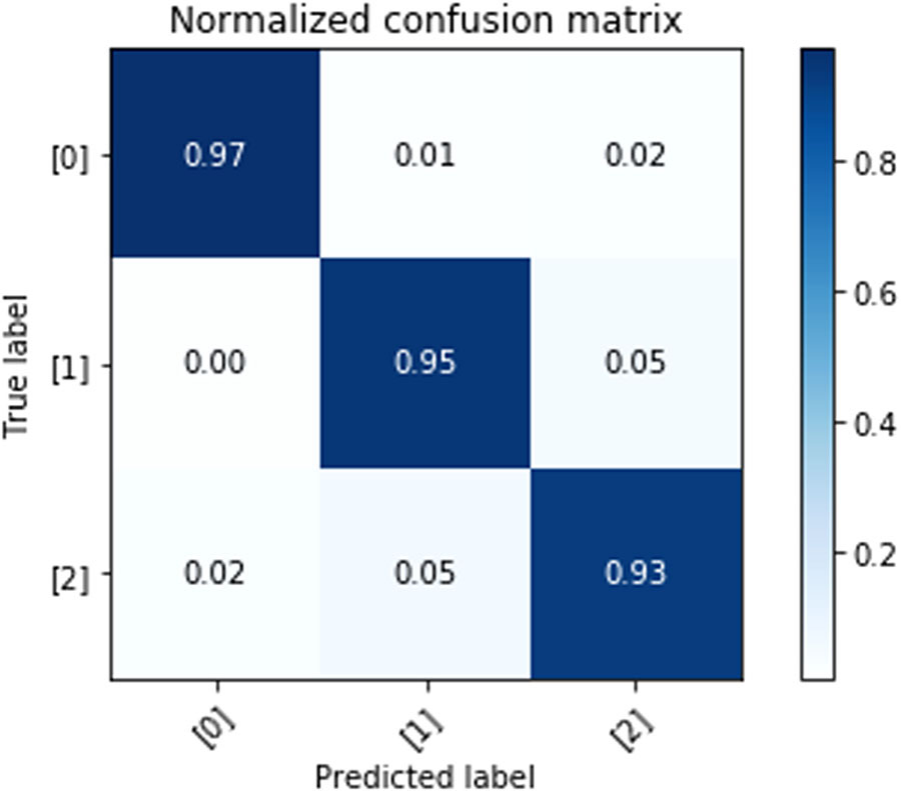
Normalized confusion matrix for STAI evaluation utilizing visual metrics and CNN-LSTM algorithm. Element value (m, n) of this matrix, and color, represent the probability of predicted STAI level n, given the ground-truth STAI level m, where m and n have labels of 0 (low), 1(intermediate/controllable), and 2 (high). The diagonal elements of this matrix correspond to correct predictions.

**Fig. 5:**
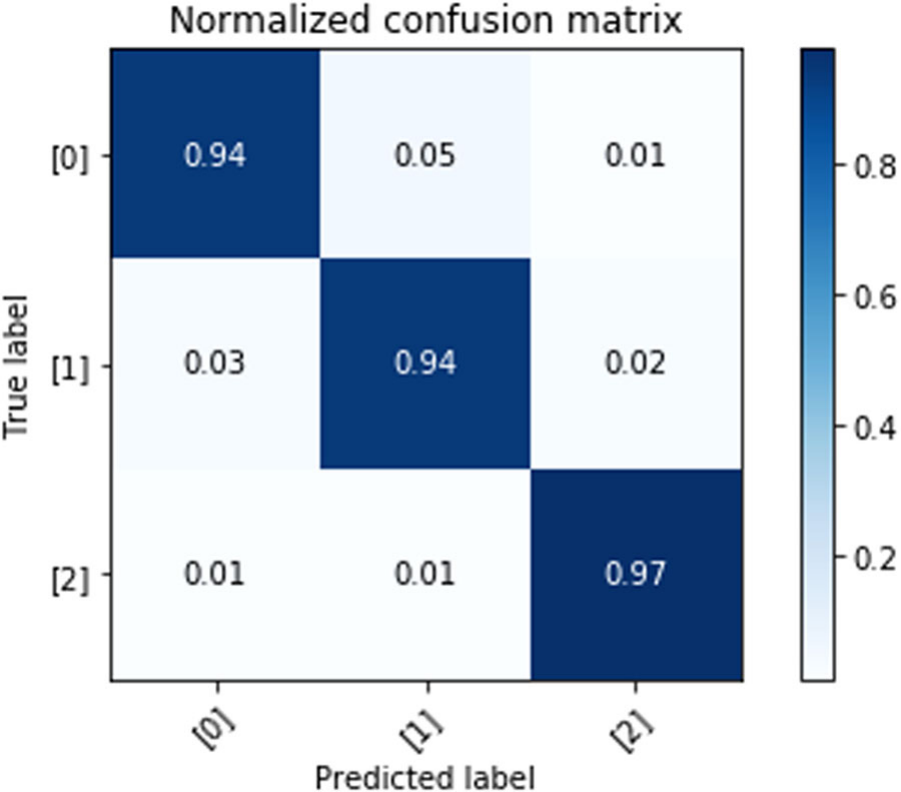
Normalized confusion matrix for WEMWBS evaluation utilizing visual metrics and CNN-LSTM algorithm. Element value (m, n) of this matrix, and color, represent the probability of predicted WEMWBS level n, given the ground-truthWEMWBS level m, where m and n have labels of 0 (low), 1(intermediate/controllable), and 2 (high). The diagonal elements of this matrix correspond to correct predictions.

We evaluate the proposed deep learning approach for self-proclaimed mental health status classification. The confusion matrices of classification results are obtained from the testing set under the five-fold LOSO cross-validation scheme. Figures 3–5 show the results of three-class self-proclaimed mental health status classification.

### Hope evaluation

The F1-score: 93.81%, and accuracy: 93.81% for HHI level classification. Confusion matrix for HHI level classification is shown in Fig. 3.

### STAI evaluation

The F1-score: 94.77% and accuracy: 94.76% (confusion matrix shown in Fig. 4).

### WEMWBS evaluation

The F1-score: 95% and accuracy: 95% (confusion matrix shown in Fig. 5).

## Discussion

In this study we used visual metrics as source of input to CNN-LSTM model to objectify the evaluation of mental health status metrics (HHI, STAI, and WEMWBS), considering participant-based responses for validated questionnaires. Results showed promising accuracy 93.81%, 94.76%, and 95.00% for HHI, STAI, and WEMWBS, respectively. Our primary goal was to introduce and evaluate the applicability of a deep learning-based approach for objective evaluation of mental health in patients after major oncologic surgery.

We developed an end-to-end algorithm and integrated temporal dynamics on rich representations of visual time series, while simultaneously classifying mental health. LSTM has the characteristic of resolving the problem of vanishing gradients, which is a problem of RNNs^48^. LSTM introduces the concept of a memory unit. They can decide when to forget and when to remember hidden states for future time steps. Hence, LSTMs are able to train models on long term dependencies^49^. Characteristics of CNN and LSTM algorithms make their combination suitable for mental health evaluation using visual metrics time series. We recorded visual metrics in an acceptable and patient-friendly approach that would pose minimal or no additional burden or discomfort for patients after surgery. Most studies for mental health evaluation rely on subjective evaluations in combination with physiological parameters. The proposed evaluation method is end-to-end and does not require features engineering and preprocessing. The developed model may be used for formal evaluation or assessment at home to objectively monitor patient’s mental health without the need for a hospital visit in the future.

Despite the uniqueness of this study, several limitations exist. Subjective mental health metrics evaluations were based on scores given at the end of each session (observing 18 art pieces). The effect on mental health because of a particular artwork was not considered. The small number of participants is another limitation. The developed model lacks rigorous testing and evaluation for medical application. However, we believe it has the potential to monitor mental health in patients after oncologic surgery in an objective fashion, with minimal discomfort to patients.

## Conclusion

A novel data-driven deep architecture model for the objective classification of mental health metrics was developed. This model, once validated, will serve as a real-time, patient-friendly model of mental health assessment. Future work would include further validation of the model for objective and remote monitoring of mental health, as well as investigating the effect of the specific surgery type or artwork viewed.
